# A virtual climate library of surface temperature over North America for 1979–2015

**DOI:** 10.1038/sdata.2017.155

**Published:** 2017-10-17

**Authors:** Sergey Kravtsov, Paul Roebber, Vytaras Brazauskas

**Affiliations:** 1 Department of Mathematical Sciences, University of Wisconsin-Milwaukee, Milwaukee, WI 53217, USA

**Keywords:** Atmospheric science, Natural hazards, Climate and Earth system modelling

## Abstract

The most comprehensive continuous-coverage modern climatic data sets, known as reanalyses, come from combining state-of-the-art numerical weather prediction (NWP) models with diverse available observations. These reanalysis products estimate the path of climate evolution that actually happened, and their use in a probabilistic context—for example, to document trends in extreme events in response to climate change—is, therefore, limited. Free runs of NWP models without data assimilation can in principle be used for the latter purpose, but such simulations are computationally expensive and are prone to systematic biases. Here we produce a high-resolution, 100-member ensemble simulation of surface atmospheric temperature over North America for the 1979–2015 period using a comprehensive spatially extended non-stationary statistical model derived from the data based on the North American Regional Reanalysis. The surrogate climate realizations generated by this model are independent from, yet nearly statistically congruent with reality. This data set provides unique opportunities for the analysis of weather-related risk, with applications in agriculture, energy development, and protection of human life.

## Background & Summary

State-of-the-art numerical weather prediction models are expensive to run and are subject to biases due to imperfect physical parameterizations of unresolved processes^1^. An alternative strategy for weather and climate prediction, which complements a more traditional data assimilation approach (see, for example, ref. 2), builds on extremely numerically efficient empirical stochastic models^3^, which have recently been shown to be able to capture detailed spatiotemporal statistics of select climatic fields of interest^4^. In this work, we apply the latter technique to obtain and record the output from a large ensemble of surface atmospheric temperature (SAT) simulations over North America. These simulations can be used for a wide variety of probabilistic applications, for example—to estimate long-term changes in the space/time distribution and magnitude of extreme heat waves and cold spells in the region.

Modeling and predicting temperature extremes is a task of utmost societal and economic importance, especially in the context of potential changes in the extreme weather associated with global warming^5,6^. The main problem with robust detection of such changes stems from an inherent rarity of extreme events coupled with a limited length of both the observational record and comprehensive high-resolution climate-model simulations^7,8^. A standard way of dealing with these issues has been rooted in the generalized extreme value (GEV) theory^9^. In this approach, the GEV distribution—or generalized Pareto (GP) distribution—are fit to the sampled extreme values, with possible trends in the extremes accounted for by time-dependent covariates affecting the distribution parameters^10–13^. A recent remarkable study ref. 14 developed an alternative and, in a sense, more general approach to analyzing the extreme-event data using the so-called stochastically generated skewed (SGS) distributions^15,16^. The SGS distributions are fit to the entire available time series—rather than to extreme values only,—which allows one to reduce sampling uncertainties associated with a limited climate record. Importantly, these SGS distributions are generated by a corresponding damped linear Markov-process model that involves both additive and multiplicative (state-dependent) stochastic noise. It is the latter dependence that generally makes the SGS distributions non-Gaussian, which has profound implications for robust detection of change in the heavy tails of the observed distributions, and, hence, in the extreme event statistics^14^.

The stochastic modeling methodology employed in the present work can be viewed as a multivariate extension of the SGS-distribution generating stochastic model and has a long history of its own, starting from the linear inverse modeling (LIM)^17–20^, to its multi-level and nonlinear extensions^21^, to the modifications involving multiplicative noise introduced via state-dependent noise sampling^22,23^. Traditionally, the emphasis of such statistical modeling was on identifying an optimal low-order model that would be able to faithfully mimic certain features of the observed low-frequency climate variability^3^. More recently, however, an intermediately sized empirical model^24^ demonstrated skill in reproducing various aspects of high-dimensional air-sea interaction over the Southern Ocean. Testing the limits of empirical data modeling, ref. 4 adopted and modified the basic methodology ref. 21 to develop a high-resolution empirical stochastic model capable of emulating and predicting detailed variability of the Northern Hemisphere sea-level pressure over a wide range from sub-daily to inter-annual time scales. The latter proof-of-concept study provides the direct foundation for our present work.

**Figure 1:**
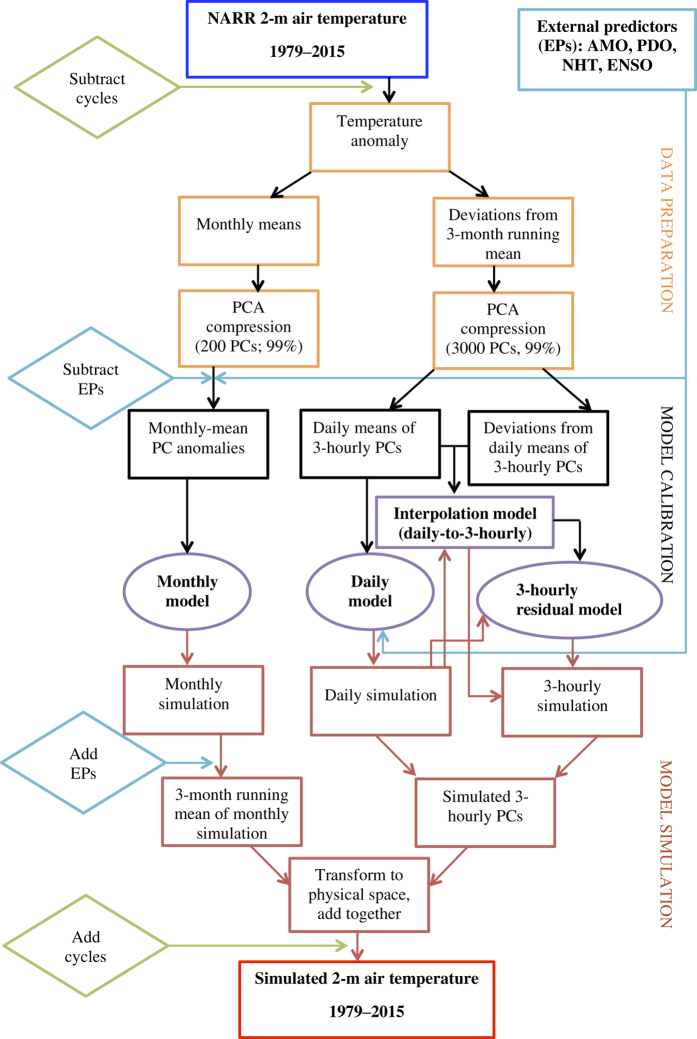
Empirical model construction and air-temperature simulation flowchart. The three-tier empirical model is based on the North American Regional Reanalysis (NARR) 2-m air temperature data and is conditioned on external predictors (EPs) that describe climate variations associated with large-scale climate modes.

Our updated empirical SAT modeling framework uses the input 2-m air-temperature data from the North American Regional Reanalysis^25^ over the 1979–2015 period to fit a multi-scale parametric statistical model for the one-way coupled sub-modules operating at different time resolutions and incorporating non-stationary dependencies on seasonal and external predictors reflecting the large-scale climate signals^12^ (Fig. 1; see Methods for details). At the simulation stage, the coupled empirical model is driven by state-dependent spatially correlated noise to provide statistical realizations of the observed SAT over the period of reanalysis; these realizations closely mimic the spatiotemporal structure of the observed SAT evolution. Accounting for the spatiotemporal dependencies within the data allows the present spatially extended multi-scale model to achieve a more robust estimation of the extreme-event statistics compared to the univariate methodologies; cf. refs 26–28. The final output climate library contains 100 independent realizations of the daily minimum and daily maximum SAT evolution over North America for the 1979–2015 period. This library, in addition to its obvious scientific value, can be used to assess the diverse extreme weather statistics^29^ that are highly relevant for applications in the agriculture, energy development and protection of human life.

## Methods

In the following description, please refer to Fig. 1, which summarizes the entire sequence of steps in model construction and subsequent simulation. Our present empirical modeling framework is largely based on the methodology developed in ref. 4.

### Empirical model input data

To build our empirical model, we used 2-m (surface) air temperature (hereafter SAT) data set based on the National Center for Environmental Prediction North American Regional Reanalysis (NARR) (http://www.esrl.noaa.gov/psd/data/gridded/data.narr.html). The NARR data set is comprised of 3-hourly (8 per day) ‘observations’ on a 349×277 grid with nominal spatial resolution of 32 km, over the 1979–2015 period; about quarter of these data are from locations within continental US, leading to ~25,000 data points in each of the ~365 (days per year)×8 (observations per day)×37 years~108,000 available SAT maps (the whole SAT data set over US thus contains ~10 Gb of single-precision data). We also utilized four external predictors, or covariates (Table 1), to represent the effects of forced and internal large-scale climate variability on weather statistics over the North America; e.g., ref. 12. We removed the mean seasonal cycle from all of the external predictor 1979–2015 time series and regressed out the ENSO signal from NHT, AMO and PDO predictors (by transforming the NHT, AMO, and PDO time series to regression residuals with respect to ENSO), then centered and normalized the resulting four time series. We also removed, via linear regression, the NHT signal from the AMO time series. This procedure resulted in the set of four nearly uncorrelated external predictors for the use with the empirical SAT model.

We first computed the SAT climatological seasonal and diurnal cycles by applying 10-day running mean to each of the 8 daily observations and averaging over all years, and formed SAT anomalies by subtracting these cycles from the raw data. Our model was built in the phase space of SAT anomalies’ empirical orthogonal functions (EOFs^30^). The EOFs were computed for the monthly-mean SAT anomalies and—separately—for high-pass filtered data set obtained by forming the deviations of SAT anomalies with respect to their three-month running mean (the latter data set had the original 3-hourly resolution); 200 monthly EOFs and 3,000 3-hourly EOFs account for over 99% of the total variance in the respective multivariate time series (herafter, we will use a generic notation **x** for the vector time series of the principal components (PCs) associated with each type of EOFs). We further subtracted the external predictors from monthly PCs by using multiple linear regression of each of the PCs onto the external predictors and only retaining the resulting PC residuals unexplained by the regression fit (these residuals are, by construction, uncorrelated with external predictors). We also split the three-hourly PCs into the daily-mean component and 3-hourly deviations from the daily means. We now look to construct a multi-scale empirical model able to provide a faithful statistical simulation of each ‘tier’ of the data—monthly, daily and three-hourly—and to capture possible interactions between these tiers.

### Model calibration

The model’s building block is a stochastic autoregressive-moving average (ARMA) model for the principal components **x**, postulated to have the following multi-level form^21^:

(1)dx=x⋅A(1)+r(1),dr(1)=[r(1)x]⋅A(2)+r(2),dr(2)=[r(2)r(1)x]⋅A(3)+r(3).

Here d**x**=**x**^n+1^−**x**^n^, and n is the time index; note that state vectors and residuals on the right-hand side of (1) are all estimated at time n. The model’s parameters (linear operators **A**, as well as each level’s residuals **r**) are found via regularized multiple linear regression and depend on seasonal cycle at monthly resolution; at the simulations stage, the last level residuals **r**^(3)^ are modeled stochastically. The multi-layer stochastic model (1) can be viewed as a way of enlarging the phase space to unravel the dynamical memory kernel of the system; see work ref. 31 for theoretical perspective on this approach using the Mori-Zwanzig formalism.

The model (1) was estimated separately for temperature time series at monthly, daily and three-hourly resolutions. For modeling the latter three-hourly anomalies (with respect to daily means), we developed a diagnostic interpolation model to isolate the part of this three-hourly variability directly tied to the synoptic daily variability (‘Interpolation model’ in Fig. 1; see ref. 4 for details), and only used one model level in (1), which corresponds to the classical LIM model^17–20^, to model the remaining part of the 3-hourly variability. For the daily model, we also estimated the linear relationship between the monthly variance of the third-level residual variability **r**^(3)^ and external predictors; we then computed and saved the time series of the time-dependent amplitude of **r**^(3)^ associated with external predictors, as well as the library of the normalized residual variability ${r\prime }^{\left(3\right)}$ void of this dependence.

The daily component of the empirical stochastic model developed here thus has three levels, and its three-hourly component has only one level. These choices were justified by analyzing the lag-covariance structure of the respective model residuals (see Supplementary File 1). In particular, Supplementary Fig. 1 displays the values of lag-1 autocorrelation of the residuals at the last level of the respective models. The third-level residuals of the daily model are indeed white, since their lag-1 autocorrelations are statistically indistinguishable from zero (Supplementary Fig. 1a). By contrast, the three-hourly model’s residuals are not white (Supplementary Fig. 1b) and thus have decorrelation time scales that exceed three hours (but are still less than a day; not shown). The quasi-random forcing for the three-hourly model at the simulation stage comes in the form of daily snippets (of length 8, with three-hourly resolution) taken from the original library of the three-hourly model residuals on the day in which the original (observed) *daily* state is closest to the current simulated *daily* state (see ‘Stochastic simulation’ section below). This procedure accounts for non-whiteness of the stochastic forcing in the three-hourly LIM model, whose integration simultaneously ensures smoothness of the simulated three-hourly PC anomalies.

### Stochastic simulation

The monthly model was driven by the Gaussian spatially correlated white noise with covariance structure derived from the third-level residual **r**^(3)^, and the final simulation of monthly PCs was obtained by adding the previously computed part of the monthly variability linearly related to the external predictors. Thus, the monthly model simulation is independent of the daily and three-hourly simulations; however, only the three-month running mean of the monthly simulation is used in the final combined simulation product, with any additional month-to-month variability arising from the sampling associated with the variability in the daily model output. The choice of the three-month averaging time scale was subjective, dictated by our attempt to seamlessly combine the low-frequency, forced or internal, variability simulated in the monthly model, and ultra-low-frequency month-to-month internal variability arising in the daily model. The latter internal variability can have persistent regimes with time scales on the order of but not much longer than one month; hence, three-month averaging of the monthly model’s output was used to avoid ‘double counting’ of this variability in the combined multi-scale model output.

The daily and three-hourly models are one-way coupled so that the daily simulation has to be carried out first. This simulation is driven by state-dependent forcing snippets randomly pulled from the library of the normalized third-level residuals ${r\prime }^{\left(3\right)}$ based on the proximity of the current simulated state **x**_**S**_ to a small subset of the observed states **x**; the idea borrowed from refs 22,23. The forcing so computed is then scaled to have the appropriate amplitude as per the amplitude dependence on the external predictors. The three-hourly simulation consists of two parts: the part diagnostically related to the simulated daily PCs through the interpolation model, and the remainder, simulated using a one-level LIM model. The stochastic forcing for this LIM model is also conditioned on the simulated daily state, in ways analogous to the forcing specification for the daily model. Combining the daily and hourly simulations produces the final realization of the full 3-hourly PCs.

The three-month running mean of monthly simulation and full three-hourly simulation are then transformed from the EOF phase space back to physical space, combined and, upon addition of seasonal climatology and diurnal cycle, provide the final emulation of the observed SAT variability. We produced 100 such simulations of 1979–2015 SAT. The final data product contains the output for daily maximum and daily minimum SAT time series for each of these simulations (Data Citation 1).

### Code availability

All annotated MATLAB © code and supporting input/output data for the present empirical SAT model are also available from Figshare Data Repository (Data Citation 1). This archive contains detailed README text files that would allow the user to reproduce all of the data preparation, model construction and simulation steps outlined in the flowchart of Fig. 1. In particular, one can easily obtain, if necessary, additional independent realizations of the 1979–2015 SAT evolution, which may be especially useful for quantifying the probabilities of rare events^14^. The code archive includes the scripts for reading and plotting the data in MATLAB. A basic plotting script can also be found here in the Supplementary Script 1.

## Data Records

The model output data are available as a collection of NetCDF4 files (Data Citation 1; Table 2), which naturally incorporate all of the associated metadata; for example, see Supplementary File 2. The data archive consists of four groups, two of which document the raw (unprocessed) simulated daily maximum and daily minimum SATs. The other two are the daily maximum and daily minimum SAT versions quantile-mapped to observations^32^, so that the observed and simulated local distributions of either of these quantities at a given grid point for each season become identical by construction. To do this quantile mapping, we sorted SAT values in the observed and simulated time series at a given grid point (and for a given season) in the ascending order, and replaced the sorted simulated values with the sorted observed values, then restored the original order of the values in the simulated time series. In this quantile mapping, the four seasons were defined as December-January-February (DJF), March-April-May (MAM), June-July-August (JJA) and September-October-November (SON) seasons. Also included in the archive is the daily maximum and daily minimum SAT records based on the truncated NARR data to span the phase space of its 3,000 leading EOFs, which describe over 99% of the total SAT variance over the (land) region of simulation; this is to match the truncation applied in the empirical model simulations. Note that the quantile mapping described above was also done using the NARR data set so truncated, rather than the full original NARR SAT data.

The simulated data within each data group is further split into ten subgroups of ten simulations each, and archived in individual files for each year, with a typical file size around 400 Mb. Note that this size reflects the NetCDF4 data compression, and the actual data size would be substantially larger.

## Technical Validation

Our empirical model is not expected to produce climate realizations pathwise similar to the observed climate; rather on the contrary, the climate simulated by such a model would be, by construction, statistically independent of the actual observed climate realization. Hence, we should judge the success of the model’s performance by comparing not the pathwise convergence, but rather the long-term statistical properties of the observed and simulated variability, as in ref. 4.

We first assessed the model performance in the EOF phase space—via comparing the observed and simulated probability density functions (PDF) and autocorrelation functions (ACF) of individual PCs (see Supplementary File 1, Supplementary Figs 2–4). The observed PDFs and ACFs are mostly contained within the 95% spread of these quantities in model simulations, indicating the model’s success in capturing the observed statistics. It is generally more challenging to achieve a good statistical fit between the observations and model simulations in physical space, as the transformation of model time series into physical space may, in principle, distort the local behavior. We start by displaying a couple of illustrative examples of model simulations, alongside with the corresponding observations, to give the reader a feel for phenomenological complexity of the SAT evolution, and the model’s ability to mimic this complexity.

### Simulation examples

**Figure 2:**
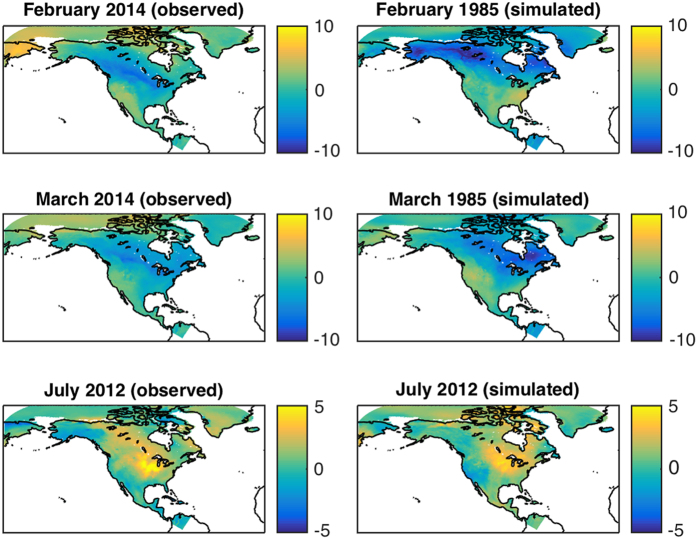
Examples of the observed and simulated monthly-mean surface air temperature anomalies with respect to the long-term monthly climatology. Left panels: observations; right panels: model simulations. The first two rows show a persistent January-February-March cold spell (January not shown); the bottom row exemplifies summertime drought conditions. Units are °C.

One of the major advantages of the empirical model considered here—compared to simpler traditional one-dimensional statistical data modeling at each spatial grid point—is that it is able to capture complex spatiotemporal relationships between the features of the temperature variability associated with forced and internal atmospheric dynamics. Figure 2 shows examples of the observed and simulated anomalous seasonal cold (top two rows) and warm conditions (bottom row). Note that the persistent cold-spell events we have chosen happen in different years in observations and model simulations, which means that they likely stem from the internal dynamics—and are tentatively due to enhanced frequency of synoptic events causing cold-air outbreaks in the months considered. On the other hand, the July 2012 anomalously warm conditions over US Great Plains happen both in observations and in model simulations, suggesting that this pattern is externally forced; cf. refs 33,34. A more in-depth analysis of ensemble simulations of the empirical model can provide further details on the contributions of forced signals and internal variability to the observed variations of the surface temperature.

Similarly to simulating month-to-month variability akin to the observed variability, the model is able to capture complex spatiotemporal behavior of temperature anomalies associated with stationary and propagating synoptic features (T2_Obs_Sim_DJF_1992_93.mov, Data Citation 1); this captured complexity is at the heart of the model’s skill in emulating the observed local weather and its long-term changes (see below).

### Distributions in physical space

**Figure 3:**
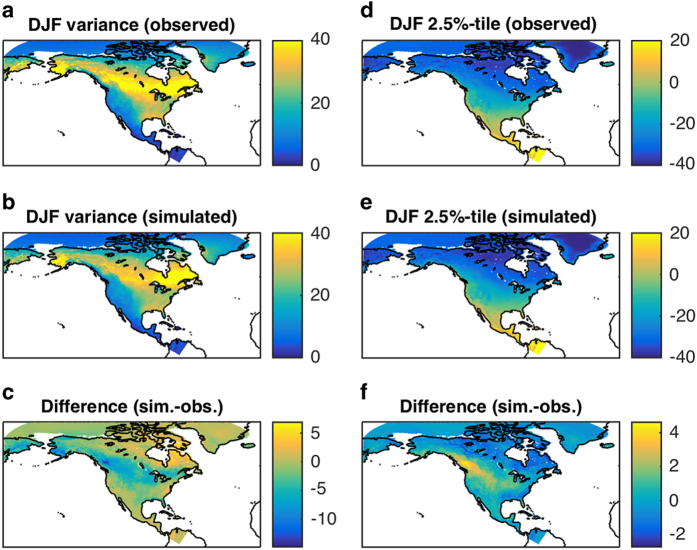
Comparison between the observed and simulated surface-temperature variability for December-January-February (DJF) period. (**a**–**c**) temperature variance (°C^2^); (**d**,**e**) extreme events represented by the 2.5th percentile of the temperature distribution (°C). Model simulations closely capture the spatial patterns of the observed variability. The most pronounced differences between observations and model simulations occur over northwestern and central North America, where the model tends to underestimate the temperature variance (**c**) and, in relation to that, the intensity of cold-air outbreaks (**f**).

The model reproduces well the seasonal cycle of temperature variance (Supplementary File 1,Supplementary Fig. 5). The largest discrepancy between the model simulated and observed variance occurs during the cold DJF season (Fig. 3a–c), where the model, while capturing very well the spatial pattern of the variability, somewhat underestimates the magnitude of this variability over northwestern and central North America, primarily due to overly diffusive (in space) cold polar air intrusions from the Arctic plains (a hint of this behavior can be seen in T2_Obs_Sim_DJF_1992_93.mov, Data Citation 1).

The model also captures quite well the spatial distribution of DJF extreme cold (Fig. 3d–f), as well as JJA extreme warm events (Supplementary File 1, Supplementary Fig. 6). There seems to be, once again, a warm bias in reproducing wintertime extreme cold conditions over the northwestern and central North America (Fig. 3f), possibly related to the variance bias detected in Fig. 3f. The JJA biases are slightly smaller than DJF biases, and have a different spatial pattern (Supplementary File 1, Supplementary Fig. 6).

Overall, the model does a fairly good job in reproducing the observed seasonal distributions of SAT, but with notable discrepancies mentioned above. To account for this, we provide, in the present data set (Data Citation 1, Table 2)—in addition to raw simulated data,—the versions of the daily minimum and daily maximum SAT time series quantile-mapped to observations^32^ (for each simulation and over the entire simulated time series; see Data Records for details).

### Dependence on external predictors

**Figure 4:**
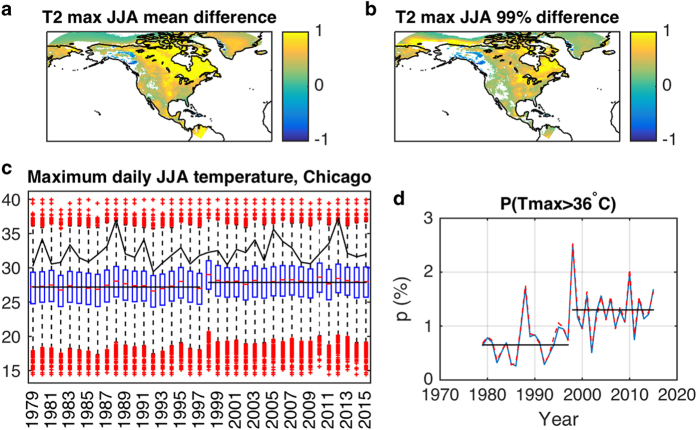
Nonstationarity of the simulated surface-temperature distributions associated with external predictors. Top row shows the difference maps (°C) between the simulated June-July-August (JJA) maximum daily surface-temperature distributions computed over the 1998–2015 and 1979–1997 periods in (**a**) mean temperature, and (**b**) 99th percentile of temperature; only the differences statistically significant at 5% level according to the 2-sided *t*-test are displayed. Bottom row shows the evolution of the JJA daily maximum surface-temperature distributions at a grid point closest to the Chicago O’Hare International Airport: (**c**) boxplot based on the entire ensemble of 100 simulations shows the interquartile range (blue box), median (red line within the box) and outliers (red plus-symbols) of temperature for each year; also shown are the 1979–1997 and 1998–2015 means of the observed temperature (horizontal black lines), as well as the 95th percentile of the observed temperature (black curve). (**d**) displays the probability of temperature to exceed the threshold based on the 99th percentile of temperature computed over the entirety of simulated data for this grid location, for the raw simulated data (blue), as well as for the simulated data quantile-mapped to observations (dashed red line). The mean 1979–1997 and 1998–2015 probabilities (horizontal black lines) differ by a factor of two.

Our empirical model incorporates the dependence on external predictors, which can affect both the mean and the variance of the simulated SAT variability (note that with fixed (constant) external predictors, the distributions simulated by the model will necessarily be stationary, aside from seasonal dependences). To demonstrate the reality of this effect, we display in Fig. 4a,b the difference maps between the simulated JJA maximum daily surface-temperature distributions computed over the 1998–2015 and 1979–1997 periods; shown are the differences in the mean and 99th percentile of these distributions, respectively. The above distributions for each year were computed based on the 100 quantile-mapped simulations (and the resulting 92 days×100 simulations=9,200 ‘time’ points for each grid location). There is a statistically significant warming across the majority of the continent, with the difference in the mean generally exceeding the difference in the 99th percentile, indicating changes in the *shape* of the SAT distribution in response to the external factors.

Figure 4c shows the time series of select characteristics of the PDF for the JJA daily maximum SAT at a grid point closest to the Chicago O’Hare’s International airport. The temperature variations exhibit quite a range of values throughout the time series, on the order of 25 °C. Interannual (year-to-year) variability and long-term trends in the surface-temperature distributions (for example, changes in its annual median or 99th percentile) are also present, but are naturally very slight compared to the overall range of variability, indicating the slowness of the climate change compared with the day-to-day SAT variations. Yet, statistically significant long-term trends in these quantities are real and noticeable (reflecting, technically, the effect of external predictors on the SAT variability in the present model’s context). A notable feature of the JJA surface-temperature behavior includes an apparent jump (of around 1 °C) to warmer conditions after year 1997, following a major El Niño episode of 1997/98. This regional climate shift may be related to a larger-scale climate shift^35^ manifested, among other things, in the flipped phase of the PDO, whose index is, incidentally, one of the external predictors forcing the present model (Table 1).

No matter how slight the latter long-term changes may seem, they are reflected in the substantial changes in the probability of extreme events. For example, the probability of the JJA maximum daily surface temperatures to exceed the 99th percentile of this quantity (computed over the entire 1979–2015 period) increases by a factor of two after the 1997/98 transition to warmer conditions (Fig. 4d). Note that the highest temperature of around 40 °C at Chicago O’Hare airport was only recorded once in the real observational record (on July 13, 1995). Yet, the empirical model simulations predict non-zero likelihood of such events over most of the 1979–2015 period, with year 1995 being, on average, less likely to produce such extreme warmth than the later periods.

The take-away point here is that the types of probabilistic information our model simulations supply cannot really be obtained based on the direct statistical analysis of the observational record, which demonstrates the essential utility of our proposed empirical modeling methodology. Needless to say that completing similar tasks using the high-resolution dynamical models (that is, numerical models based on first physical principles and state-of-the-art parameterizations of unresolved processes) is still computationally prohibitive.

## Usage Notes

The structure of the data archive is meant to facilitate obtaining certain types of probabilistic information, by allowing one to read in the data from all of the 100 available simulations for a given year. If a single continuous simulation would be of interest instead, it would probably be easier to read it in from the ensemble-simulation files year-by-year and store each year in a temporary directory for further analysis of this simulation.

## Additional Information

**How to cite this article:** Kravtsov, S. *et al.* A virtual climate library of surface temperature over North America for 1979–2015. *Sci. Data* 4:170155 doi: 10.1038/sdata.2017.155 (2017).

**Publisher’s note:** Springer Nature remains neutral with regard to jurisdictional claims in published maps and institutional affiliations.

## Supplementary Material



Supplementary Figures

Supplementary Information

Supplemental Script 1

## Figures and Tables

**Table 1:** External predictors (monthly-resolution time series) used in empirical model construction.

**Predictor name**	**Acronym**	**Source**
Northern Hemisphere mean temperature	NHT	GISS surface temperature analysis^36,37^: https://data.giss.nasa.gov/gistemp
Atlantic Multidecadal Oscillation index	AMO	AMO^38^ unsmoothed, detrended from the Kaplan SST V2. Calculated at NOAA/ESRL/PSD1: http://www.esrl.noaa.gov/psd/data/timeseries/AMO/
Pacific Decadal Oscillation index	PDO	Data^39,40^: http://jisao.washington.edu/pdo/PDO.latest
Niño3.4 index	ENSO	Nino 3.4 Index using ersstv4 from CPC: https://www.esrl.noaa.gov/psd/data/climateindices/list/#Nina34

**Table 2:** Description of model output files included in the data release.

**Compressed Folder Name**	**File Names**	**Description**
Tmax_simul#-#.zip	air.2m.1979.ncair.2m.1980.ncair.2m.1981.ncair.2m.1982.ncair.2m.1983.ncair.2m.1984.ncair.2m.1985.ncair.2m.1986.ncair.2m.1987.ncair.2m.1988.ncair.2m.1989.ncair.2m.1990.ncair.2m.1991.ncair.2m.1992.ncair.2m.1993.ncair.2m.1994.ncair.2m.1995.ncair.2m.1996.ncair.2m.1997.ncair.2m.1998.ncair.2m.1999.ncair.2m.2000.ncair.2m.2001.ncair.2m.2002.ncair.2m.2003.ncair.2m.2004.ncair.2m.2005.ncair.2m.2006.ncair.2m.2007.ncair.2m.2008.ncair.2m.2009.ncair.2m.2010.ncair.2m.2011.ncair.2m.2012.ncair.2m.2013.ncair.2m.2014.ncair.2m.2015.nc	Simulated daily maximum temperatures for each year in NetCDF4 format, in bundles of 10 realizations stored in separate directories. The ‘#’ characters in the zipped directory name indicate the range of realizations, from 1–10 to 91–100.
Tmax_QM_simul#-#.zip		The same as above, but for the surface-temperature simulations quantile-mapped to observations
Tmin_simul#-#.zip		Simulated daily minimum temperatures for each year in NetCDF4 format, in bundles of 10 realizations stored in separate directories. The ‘#’ characters in the zipped directory name indicate the range of realizations, from 1–10 to 91–100
Tmin_QM_simul#-#.zip		The same as above, but for the surface-temperature simulations quantile-mapped to observations
Tmax_observed_nc.zip		NARR based daily maximum surface air temperature reflecting the variability associated with the 3,000 leading EOFs of the full data set. This is achieved by performing PCA decomposition of the NARR data, truncating at 3,000 EOF modes and transforming back to physical space.
Tmin_observed_nc.zip		The same as above, but for daily minimum surface air temperature.
