# Supplementary material for: A virtual climate library of surface temperature over North America for 1979–2015
Source: Sci Data. 2017 Oct 17;4:170155. doi: 10.1038/sdata.2017.155 (PMC5644371; doi:10.1038/sdata.2017.155)
Supplement: Supplementary Figures [file sdata2017155-s2.pdf]

(a)

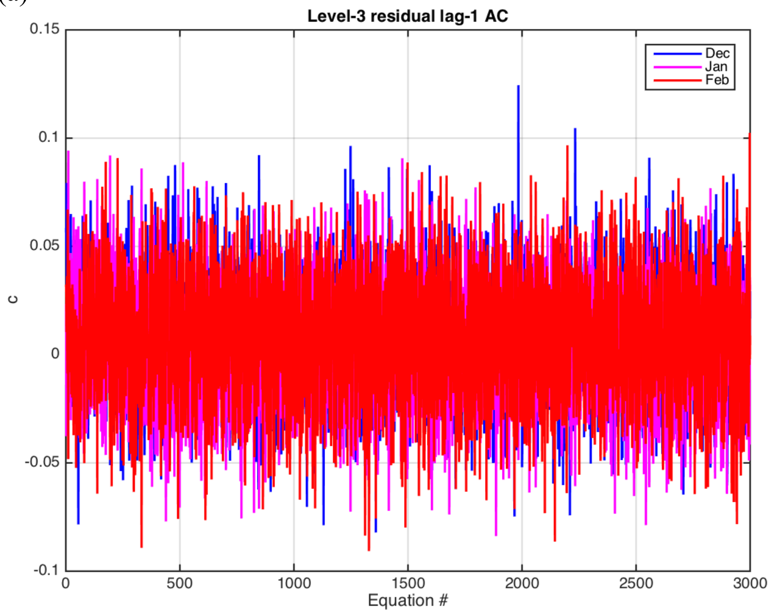

(b)

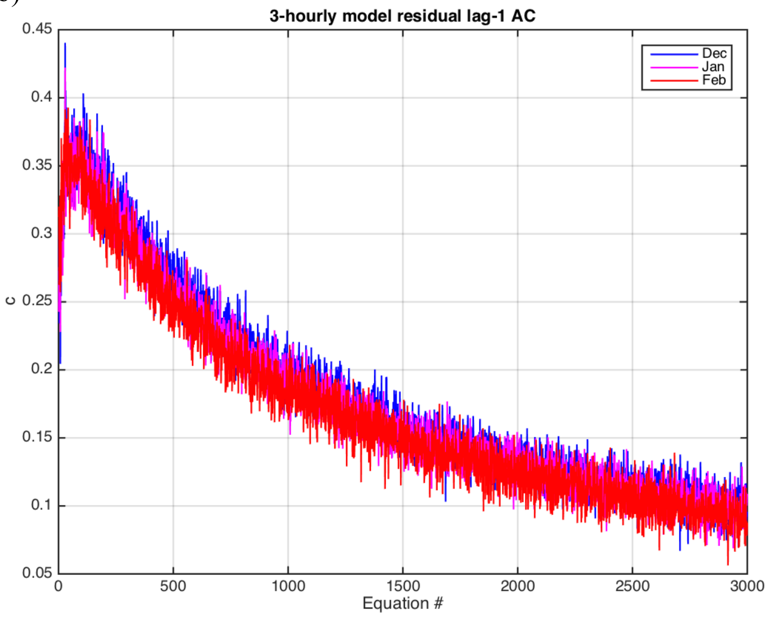

Figure S1. Lag-1 autocorrelation of the residuals in (a) the third level of the daily model; and (b) the first (and only) level of the three-hourly model. The autocorrelations are shown for the residuals in each of 3000 model equations (as marked in abscissa), for the December (blue), January (magenta) and February (red) data.

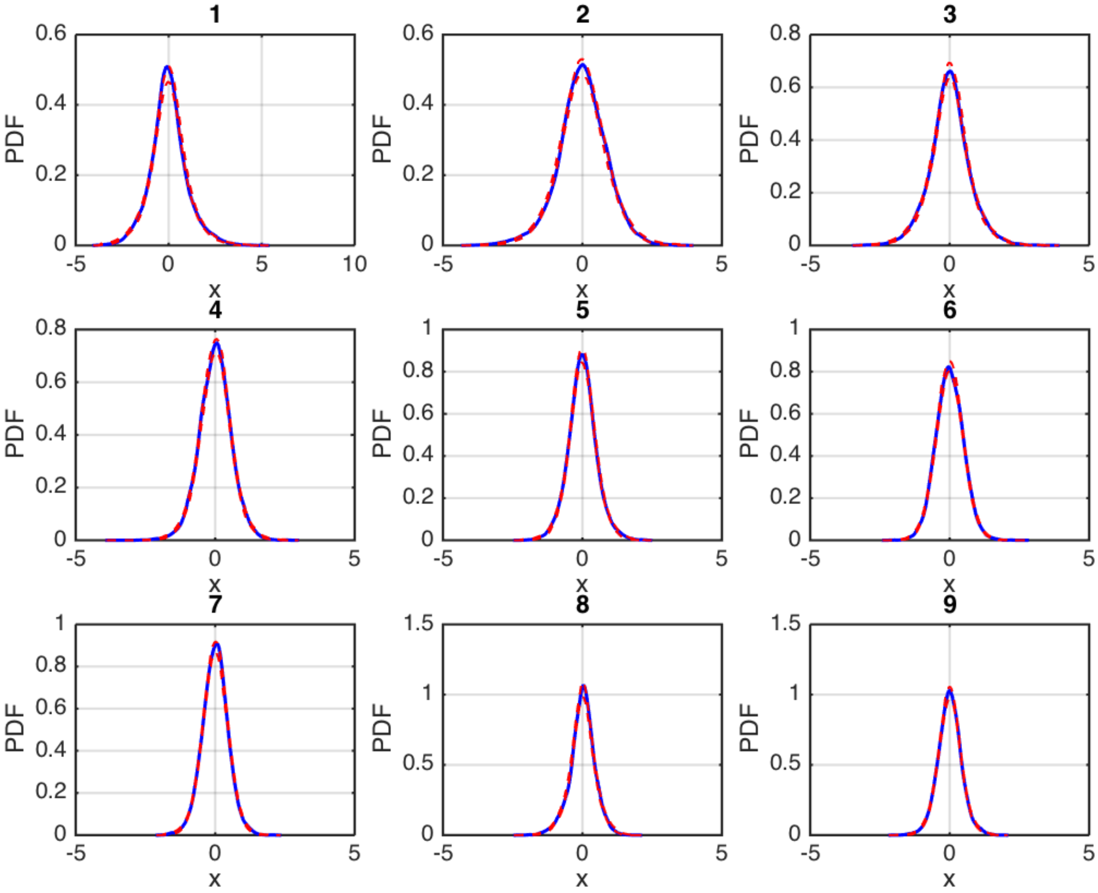

Figure S2. The probability density function (PDF) of the observed (blue) and simulated daily PCs (red dashed lines show the 95% spread over 100 simulations). Shown are the results for 9 leading PCs.

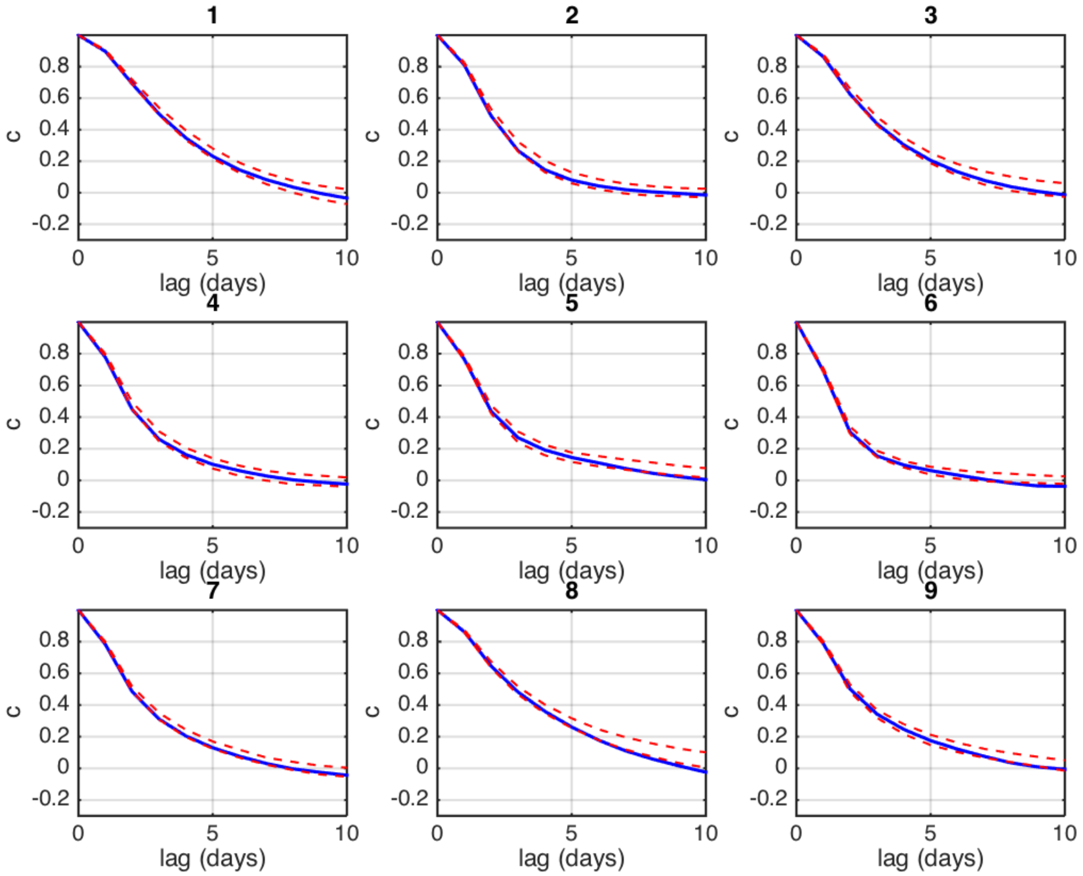

Figure S3. The autocorrelation function (ACF) of the observed (blue) and simulated daily PCs (red dashed lines show the 95% spread over 100 simulations). Shown are the results for 9 leading PCs.

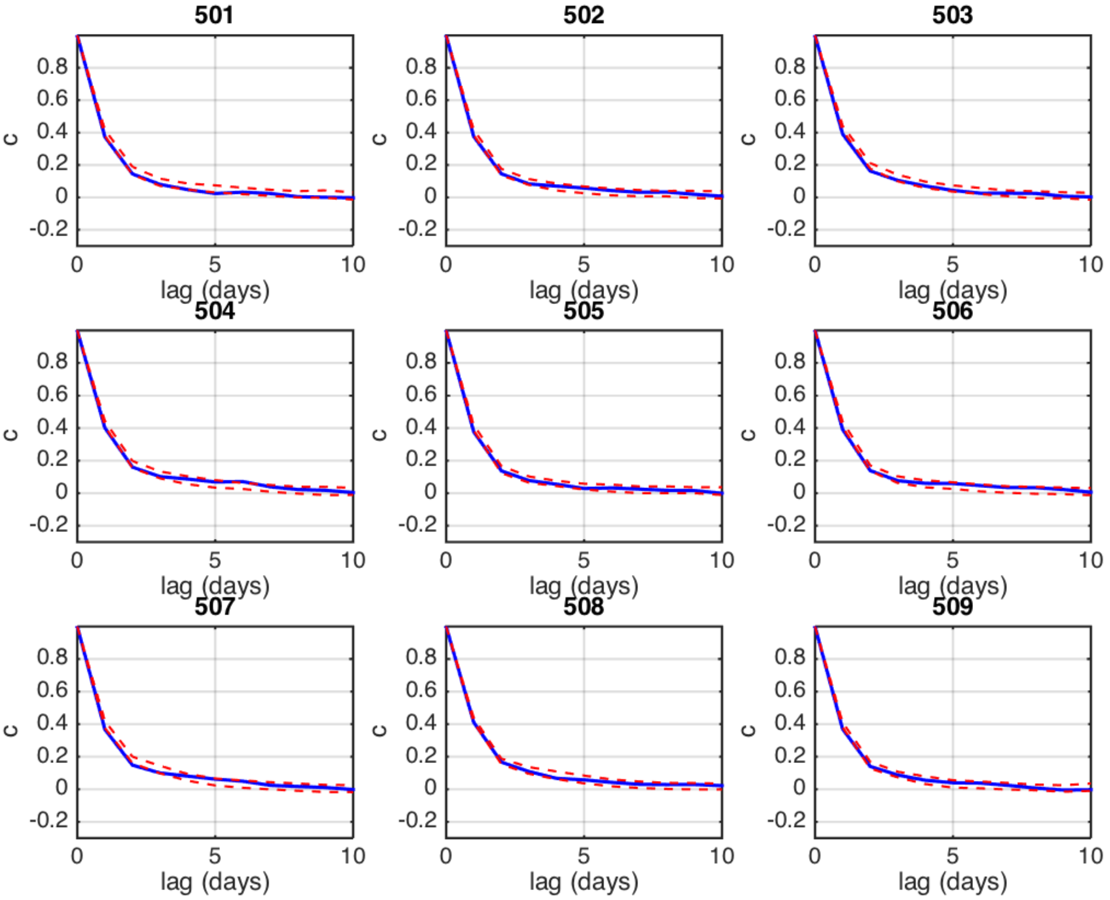

Figure S4. The same as in Fig. S3, but for the observed and simulated PCs 501–509.

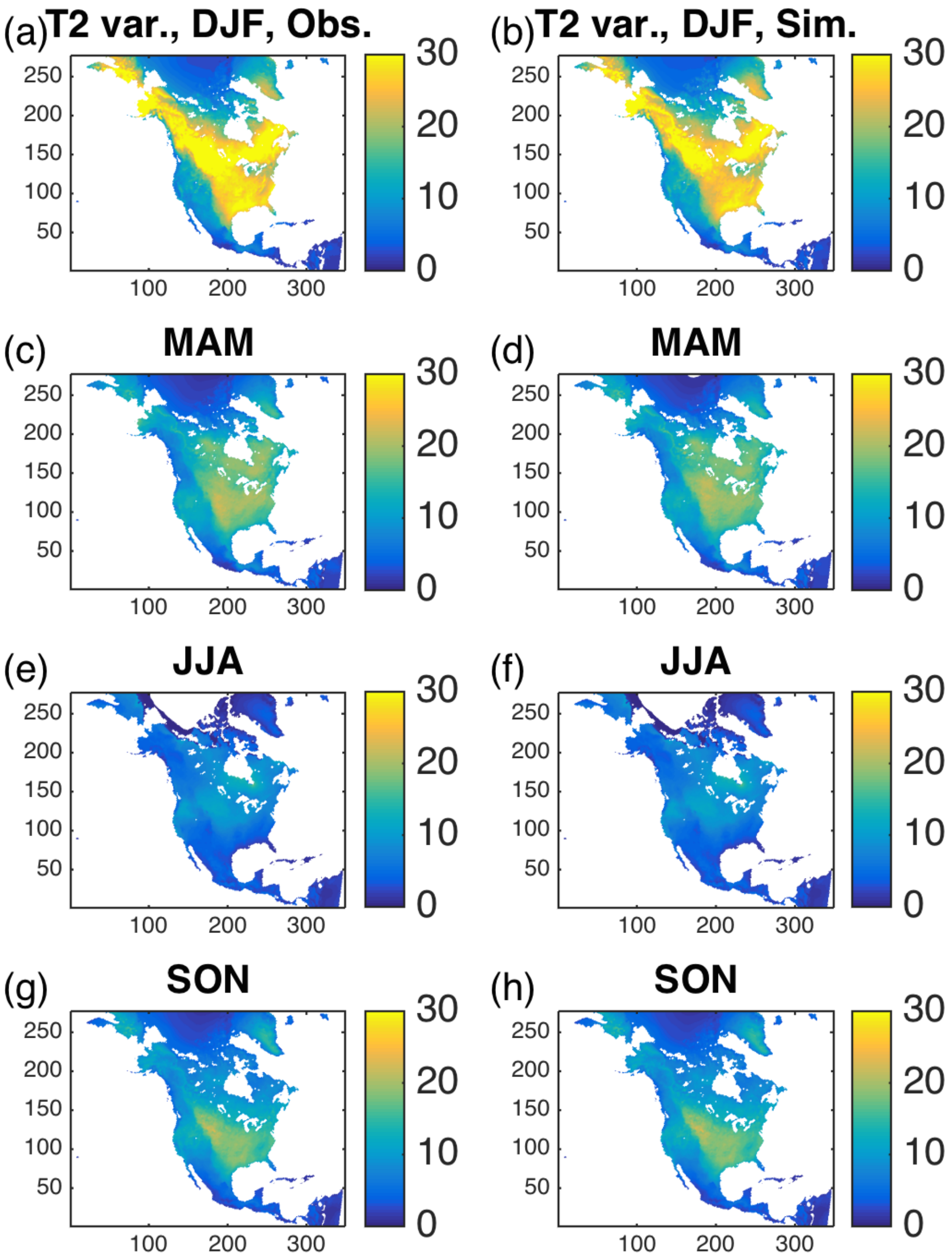

Figure S5. Seasonal cycle of temperature variance in 1979–2015 observations (left) and a model simulation (right). Spatial distributions of variance are shown for December–January–February (DJF), March–April–May (MAM), June–July–August (JJA), and September–October–November (SON) seasons.

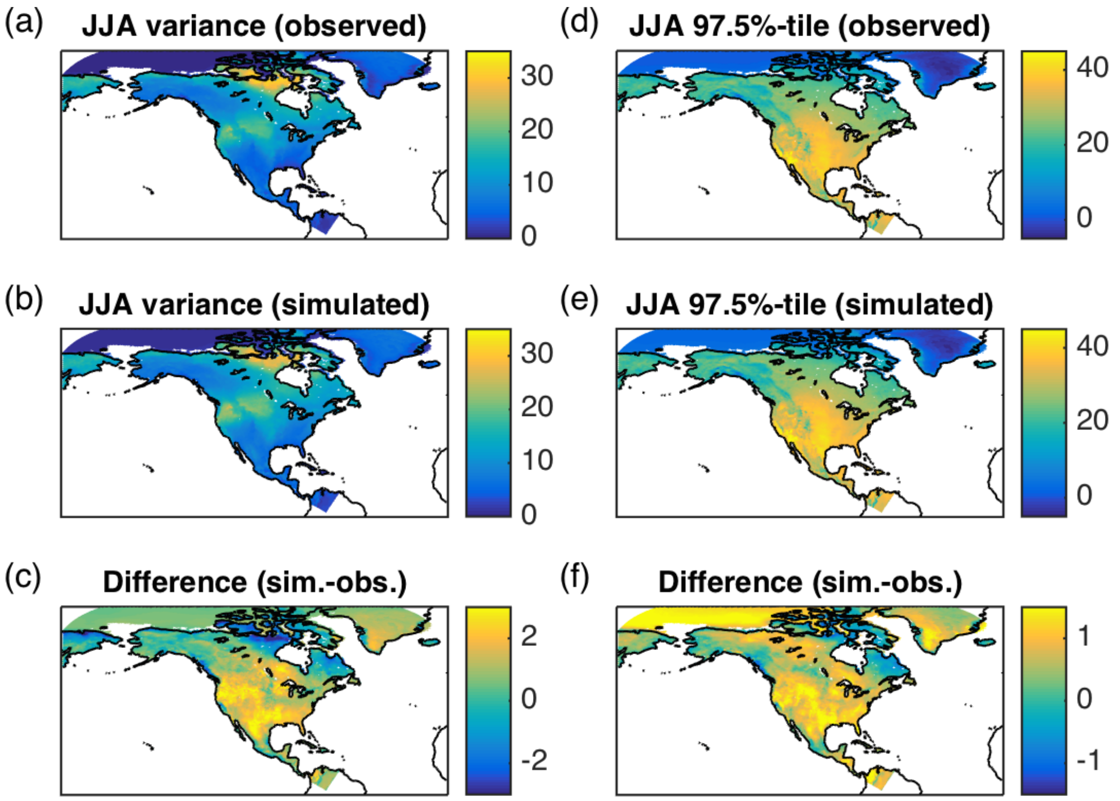

Figure S6. Comparison between the observed and simulated surface-temperature variability for June–July–August (JJA) period: (a–c) temperature variance ($^{o}C^{2}$); (d–e) extreme events represented by the 97.5th percentile of the temperature distribution (^{o}C). Model simulations capture well the spatial patterns of the observed variability, although some biases are still present.
