# Supplementary material for: A virtual climate library of surface temperature over North America for 1979–2015
Source: Sci Data. 2017 Oct 17;4:170155. doi: 10.1038/sdata.2017.155 (PMC5644371; doi:10.1038/sdata.2017.155)
Supplement: Supplementary Information [file sdata2017155-s3.pdf]

```
Source:
        /Users/kravtsov/Desktop/NARR_T2_SIMULATION/Tmax_simulated_nc/Tmax_simul1-10/air.2m.1979.nc
Format:
        netcdf4_classic
Global Attributes:
        creation_date = '17-Apr-2017 11:26:57'
        centerlat     = 50
        centerlon     = -107
        comments      = 'Produced by a statistical model trained on the NARR data for 2-m air temperature'
        institution   = 'University of Wisconsin-Milwaukee'
        latcorners    = [1     0.897945      46.3544      46.6343]
        loncorners    = [-145.5     -68.32005     -2.569891      148.6418]
        platform      = 'Statistical Model'
        references    = 'Kravtsov, Roebber, Brazauskas, 2017: Submitted to Nature Scientific Data'
        standardpar1  = 50
        standardpar2  = 50
        title         = 'Daily Max 2-m air temperature'
        dataset_title = 'Daily maximum surface air temperature over land (North America)'
        Realizations  = '1-10'
Dimensions:
        x           = 349
        y           = 277
        time        = 365
        realization = 10
Variables:
    x
        Size:       349x1
        Dimensions: x
        Datatype:   single
        Attributes:
                    long_name     = 'eastward distance from southwest corner of domain in projection coordinates'
                    standard_name = 'projection_x_coordinate'
                    units         = 'm'
    y
        Size:       277x1
        Dimensions: y
        Datatype:   single
        Attributes:
                    long_name     = 'northward distance from southwest corner of domain in projection coordinates'
                    standard_name = 'projection_y_coordinate'
                    units         = 'm'
    time
        Size:       365x1
        Dimensions: time
        Datatype:   double
        Attributes:
                    axis               = 'T'
                    coordinate_defines = 'point'
                    delta_t            = '1 day'
                    long_name          = 'Time'
                    standard_name      = 'time'
                    units              = 'days since 1979-1-1 00:00:0.0'
                    actual_range       = [1  365]
    realization
        Size:       10x1
        Dimensions: realization
        Datatype:   double
        Attributes:
                    axis               = 'N'
                    coordinate_defines = 'simulation'
```

```
            long_name         = 'Realization'
            standard_name     = 'realization'
            actual_range      = [1  10]
lon
    Size:       349x277
    Dimensions: x,y
    Datatype:   single
    Attributes:
            axis              = 'X'
            coordinate_defines = 'point'
            long_name         = 'Longitude'
            standard_name     = 'longitude'
            units             = 'degrees_east'
lat
    Size:       349x277
    Dimensions: x,y
    Datatype:   single
    Attributes:
            axis              = 'Y'
            coordinate_defines = 'point'
            long_name         = 'Latitude'
            standard_name     = 'latitude'
            units             = 'degrees_north'
Lambert_Conformal
    Size:       1x1
    Dimensions:
    Datatype:   int32
    Attributes:
            false_easting                 = 5632642.2255
            false_northing                = 4612545.6514
            grid_mapping_name             = 'lambert_conformal_conic'
            latitude_of_projection_origin = 50
            longitude_of_central_meridian = -107
            standard_parallel             = [50  50]
air
    Size:       349x277x365x10
    Dimensions: x,y,time,realization
    Datatype:   single
    Attributes:
            dataset       = 'Statistical simulation of NARR data'
            grid_mapping  = 'Lambert_Conformal'
            level_desc    = 'point'
            long_name     = 'Daily Maximum Air Temperature at 2 m'
            standard_name = 'air temperature'
            Realizations  = '1-10'
            units         = 'K'
            var_desc      = 'Air Temperature'
            missing value = -9.969209968386869e+36
            actual_range  = [227.332      325.1738]
            _Fillvalue    = 9.969209968386869e+36
```
